# Halving of the meniscectomy rate and their costs in Italy: A 15‐years period analysis

**DOI:** 10.1002/ksa.12407

**Published:** 2024-08-07

**Authors:** Umile Giuseppe Longo, Alessandro Mazzola, Marco Edoardo Cardinale, Sergio De Salvatore, Ilaria Piergentili, Robert Marx, Rocco Papalia

**Affiliations:** ^1^ Fondazione Policlinico Universitario Campus Bio‐Medico Roma Italy; ^2^ Research Unit of Orthopaedic and Trauma Surgery, Department of Medicine and Surgery Università Campus Bio‐Medico di Roma Roma Italy; ^3^ Orthopedic Unit, Department of Surgery Bambino Gesù Children's Hospital Rome Italy; ^4^ CNR‐IASI, Laboratorio di Biomatematica, Consiglio Nazionale delle Ricerche, Istituto di Analisi dei Sistemi ed Informatica Rome Italy; ^5^ Department of Orthopedic Surgery Hospital for Special Surgery, Weil Cornell Medicine New York New York USA

**Keywords:** arthritis, arthroscopy, meniscal, meniscectomy, repair

## Abstract

**Purpose:**

The purpose of this study was to determine the incidence and hospitalization trends of meniscectomy in Italy from 2001 to 2016. A secondary aim was to investigate the economic burden of the disease on the national healthcare system.

**Methods:**

Data were extracted from the Italian Ministry of Health's National Hospital Discharge Reports. Diagnoses are coded according to the ICD‐9‐CM. Meniscectomy was defined by the following main procedure codes: 806, 8026 and 8145. By dividing the number of annual cases by the size of the adult population reported annually by ISTAT, incidence rates were computed.

**Results:**

Overall, 1,454,891 meniscectomies were performed in the study period between 2001 and 2016. The incidence was 178 procedures for every 100,000 Italian inhabitants. The incidence declined from 202 in 2001 to 106 in 2016. Males were the largest portion of patients undergoing surgery (68.2%). The average age of patients was 46.59 ± 15.07. A decreasing trend in length of hospital stay was observed over the study period. The annual average cost per 100,000 inhabitants was EUR 491.219 ± 122.148 with a range from EUR 291,500 ± 79.500 in 2016 to EUR 610,500 ± 166.500 in 2004.

**Conclusion:**

In Italy, the number of meniscectomies performed in the adult population has almost halved over the study period. Results of the present study in the Italian population seem to reflect how the clinical evidence basis affects surgical technique selection. The economic burden of meniscectomy is relevant in Italy with an estimated expenditure from EUR 181.861.375 to 318.257.406 between 2001 and 2016.

**Level of Evidence:**

Level III.

**Abbreviations**
ACL: anterior cruciate ligament
DRGs: diagnosis‐related groups
ICD‐9‐CM: International Classification of Diseases, Ninth Revision, Clinical Modification
ISTAT: National Institute for Statistics
OA: osteoarthritis
SDO: National Hospital Discharge Reports

## INTRODUCTION

Meniscal tears range from severe traumatic sport‐related injuries, typically in young patients, to degenerative atraumatic and less symptomatic tears, typically in older patients [11, 34].

A systematic review of the literature defined common risk factors for meniscal tears: this injury is more likely to occur in patients over the age of 60, in men, in those who perform a lot of squatting, kneeling, stairs climbing and in patients waiting longer than a year between a functional anterior cruciate ligament (ACL) damage and surgery for reconstruction [34]. Moreover, the medial meniscus typically has a two to three times greater risk of injury than the lateral meniscus [11]. Despite its relevance, there is little information available regarding the epidemiology of meniscal injury and its management. Specific indications of conservative versus surgical treatment for meniscal tears are still debated [15, 33]. Meniscectomy, meniscal repair and transplantation are currently available surgical options for meniscus injuries, with different indications [10, 30]. Similarly to meniscal tears, also meniscectomy put the patient at a higher long‐term risk of developing osteoarthritis (OA) [20, 27]. Compared to patients treated with meniscal repair, those treated with primary meniscectomy have nearly three times higher likelihood of undergoing subsequent joint replacement [31]. Moreover, adults with degenerative and nonobstructive meniscal complaints get modest advantages with partial meniscectomy [26]. For these reasons, when feasible, meniscus repair or transplantation should be attempted [5]. National researches and epidemiologic studies show how the clinical evidence basis may affect surgical technique selection. However, divergent and conflicting patterns in arthroscopic meniscus surgery were observed in three prior US studies [2, 28, 29]. Between 2007 and 2014, the percentage of patients in Japan getting meniscus repair increased quickly, whereas the percentage undergoing meniscectomy significantly decreased [16]. According to these findings, the patterns in meniscus surgery might not accurately represent the most recent research on meniscus therapy. Nevertheless, the prior research lacked comprehensive data on patient characteristics undergoing arthroscopic meniscus procedures [2, 28, 29, 36]. More research on this topic is necessary, utilizing data from various nations. It is unknown if the increased awareness of the long‐term sequelae of meniscal deficiency and the importance of meniscal saving have resulted in reduced rates of meniscectomies in the Italian population.

The current study aims to evaluate demography, clinical patients' features and trends of meniscectomy in Italy from 2001 to 2016 in the adult population. A similar analysis has been conducted by a previous study in the paediatric setting (patients between 0 and 14 years) showing a decreasing incidence trend of meniscectomy in the last decades [23]. Our hypothesis is that over time, the number of meniscectomies in the adult population would decline. A secondary aim was to conduct an economic analysis in order to assess, in terms of costs, the burden of meniscectomy on the healthcare system.

## METHODS

The analysis of present study is based on the National Hospital Discharge records (SDO), an official database provided by the Italian Ministry of Health containing data from all Italian private and public hospitals. Diagnoses are coded according to the International Classification of Diseases, Ninth Revision, Clinical Modification (ICD‐9‐CM). Meniscectomy was defined by the following main procedure codes: 806, 8026 and 8145. Individual‐level research data are available from 2001 to 2016. These anonymized data include the patient's gender, age, place of residence, hospitalization area, length of stay, primary diagnoses and primary procedures. Population data for each year from the National Institute for Statistics (ISTAT) were used to assess the incidence of meniscectomy in Italy. The incidence rates were also stratified by year, age group and gender. We established to target the study to Italian individuals over the age of 15.

All patients included in the present study had a diagnosis of meniscal pathology and underwent a meniscal procedure (806, 8026 and 8145) even though the main primary procedure code assigned was related to other knee procedures. Exclusion was applied when a diagnosis code associated with that of meniscus surgery was atypical and did not apply to the three codes 806, 8026 and 8145 according to the ICD‐9‐CM.

### Statistics

A series of descriptive statistical analyses was carried out by means of R program, a software environment for statistical computing and graphics. Frequencies and percentage for categorical variables and means and standard deviations for continuous variables. By dividing the number of annual cases by the size of the adult population reported annually by ISTAT, a legally required electronic national population registry, incidence rates were computed.

Analyses of estimated costs were based on the cost ascribed to diagnosis‐related groups (DRGs), according to Ministerial Decree (18 December 2008). In Italy, reimbursement is the same for all the procedures under DRGs, regardless of the diagnosis, the complexity of the procedure, or the patient's health status at admission. Economic reimbursement has been calculated per single year as an average between the minimum and maximum potential reimbursement value. In Italy, indeed, reimbursement varies from region to region, hence explaining these ranges of excursion.

## RESULTS

### Demographics

**Figure 1:**
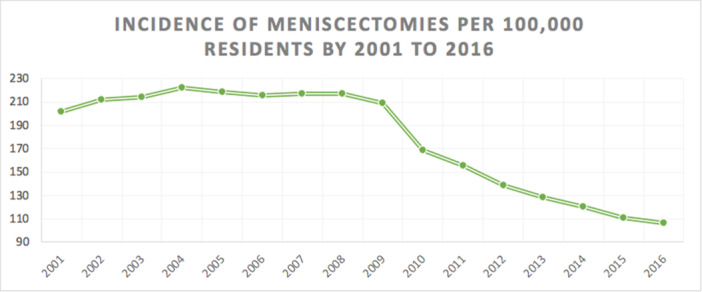
Incidence of meniscectomy (≥15 years of age) per 100,000 residents from 2001 to 2016 in Italy: the global incidence of the study was 178 per 100,000 residents; the incidence declined from 202 per 100,000 residents in 2001 to 106 per 100,000 residents in 2016.

**Figure 2:**
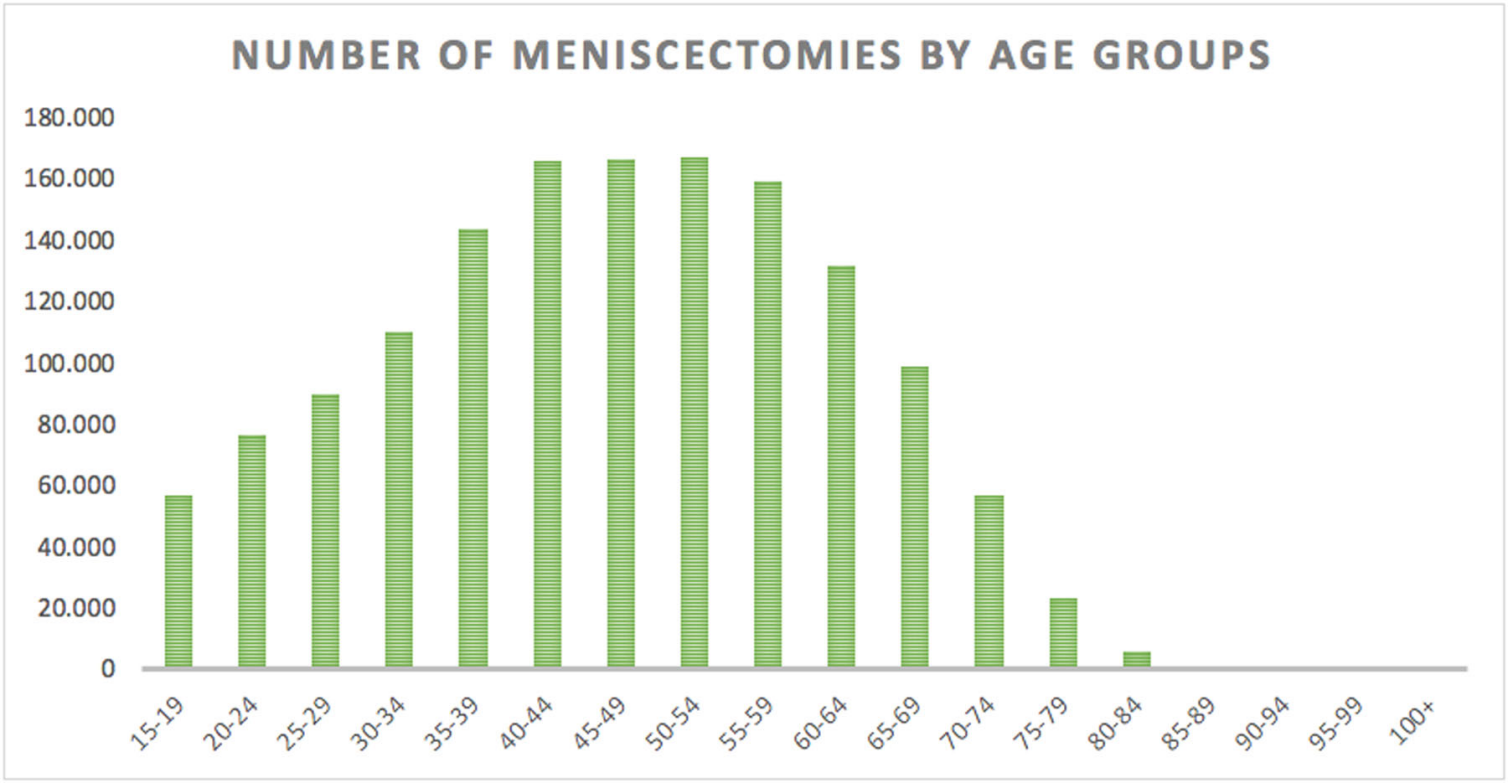
Number of meniscectomy (≥15 years of age) performed in Italy from 2001 to 2016, stratified by age groups: the age classes more representative are 40–44, 45–49 and 50–54.

**Figure 3:**
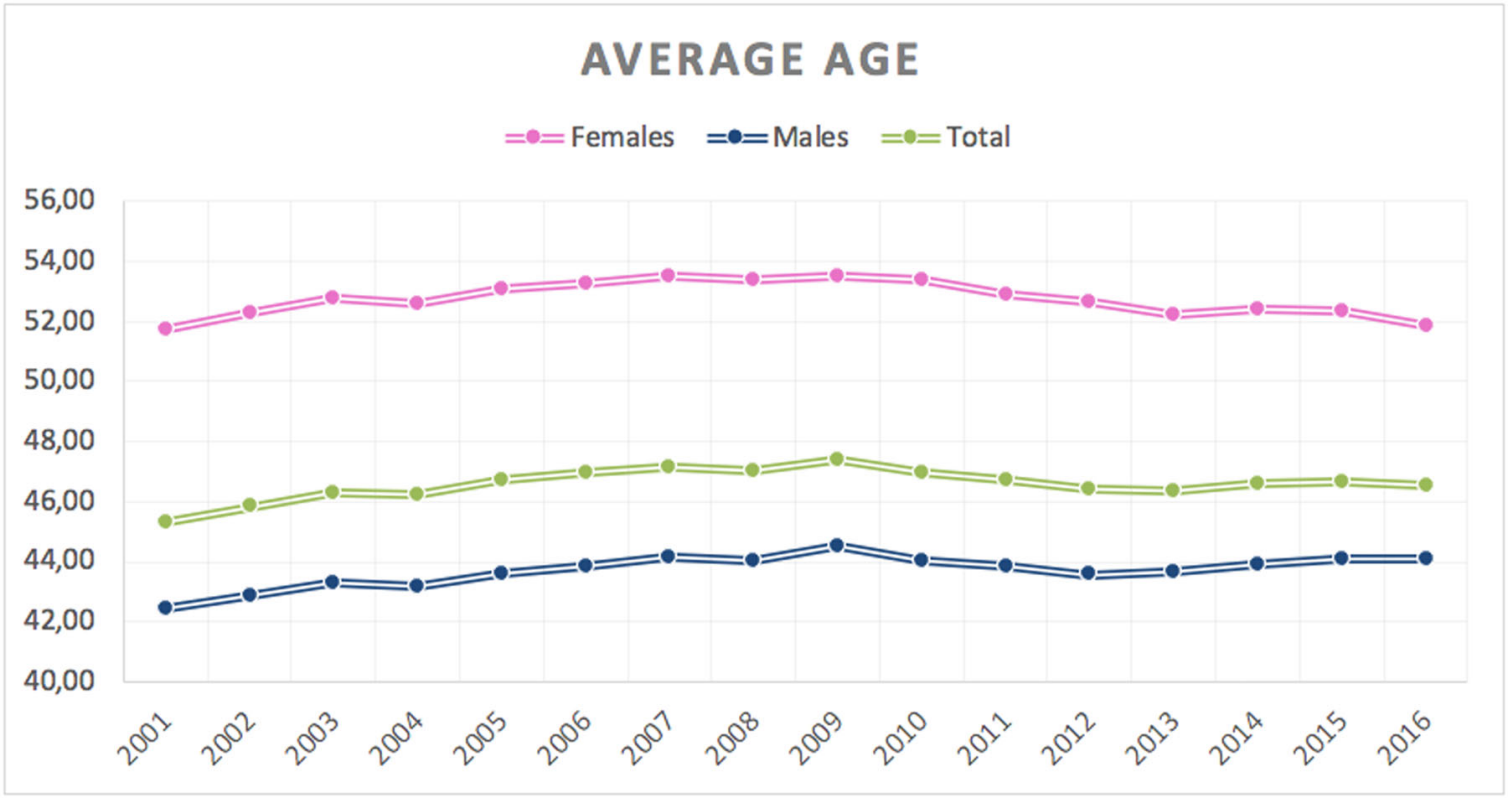
Average age of patients requiring meniscectomy (≥15 years of age) over the years by gender: males represented the majority of patients who underwent meniscectomy (females 31.8% and males 68.2%); females undergoing surgery had an average age higher than males (52.83 ± 14.53 and 43.67 ± 14.41; respectively); the average age of patients was 46.59 ± 15.07.

Overall, 1,454,891 meniscectomies were performed in the study period between 2001 and 2016. The incidence was 178 procedures for every 100,000 Italian inhabitants greater than 15 years of age. The incidence declined from 202 per 100,000 inhabitants in 2001 to 106 per 100,000 inhabitants in 2016 (Figure 1). The age classes more representative are 40–44, 45–49 and 50–54 (Figure 2). Males represented the majority of patients who underwent meniscectomy (females 31.8% and males 68.2%). In the 2001–2016 period, the average age of patients was 46.59 ± 15.07. Females undergoing surgery had an average age higher than males (52.83 ± 14.53 and 43.67 ± 14.41; respectively) (Figure 3).

### Length of the hospitalization

**Figure 4:**
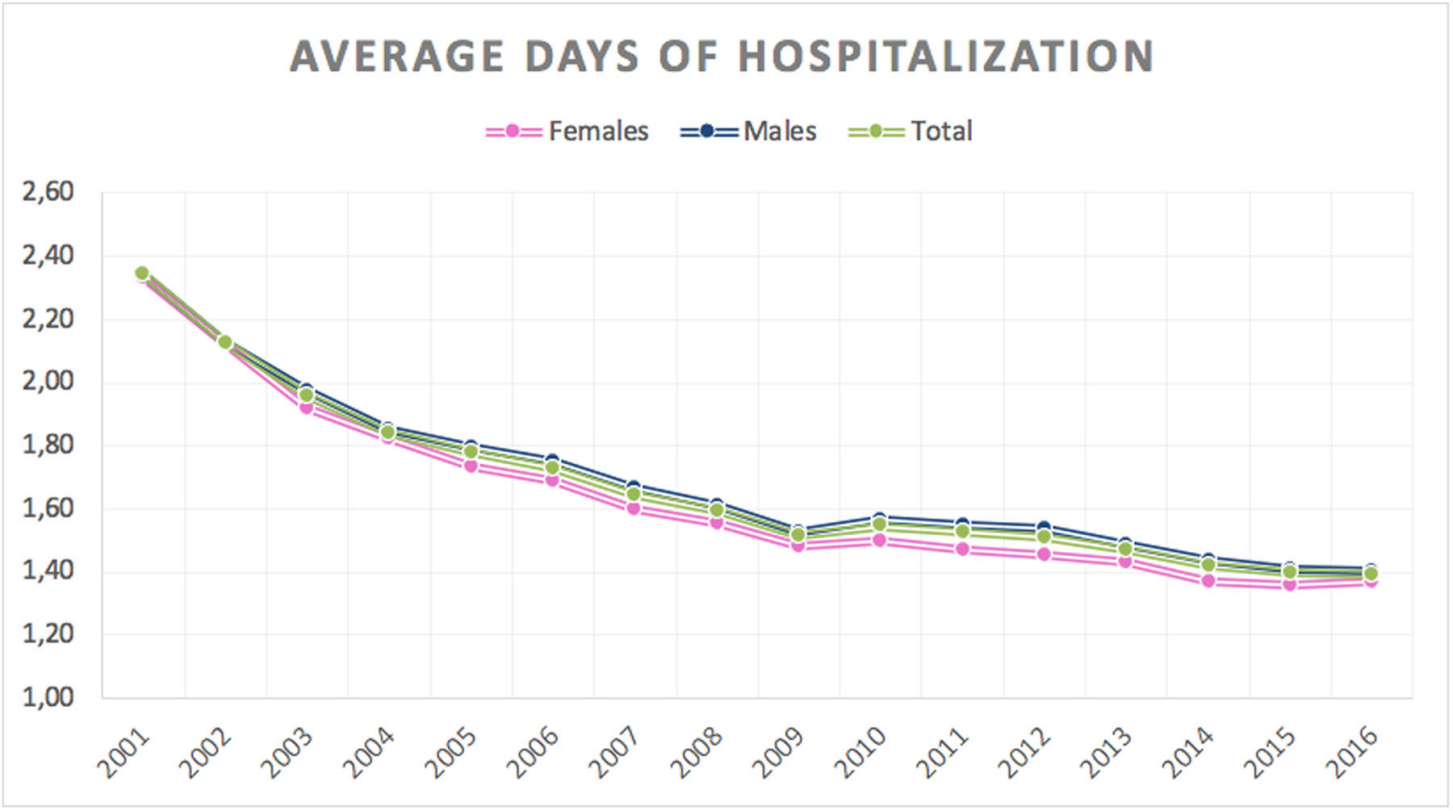
Average days of hospitalization in the study period: the average length of hospitalization was 1.71 ± 1.61 days; a decreasing trend in length of hospital stay was observed over the study period.

The average length of hospitalization was 1.71 ± 1.61 days (minimum of 0 days and maximum of 391 days). A decreasing trend in length of hospital stay was observed over the study period (Figure 4).

### Main primary diagnoses

**Figure 5:**
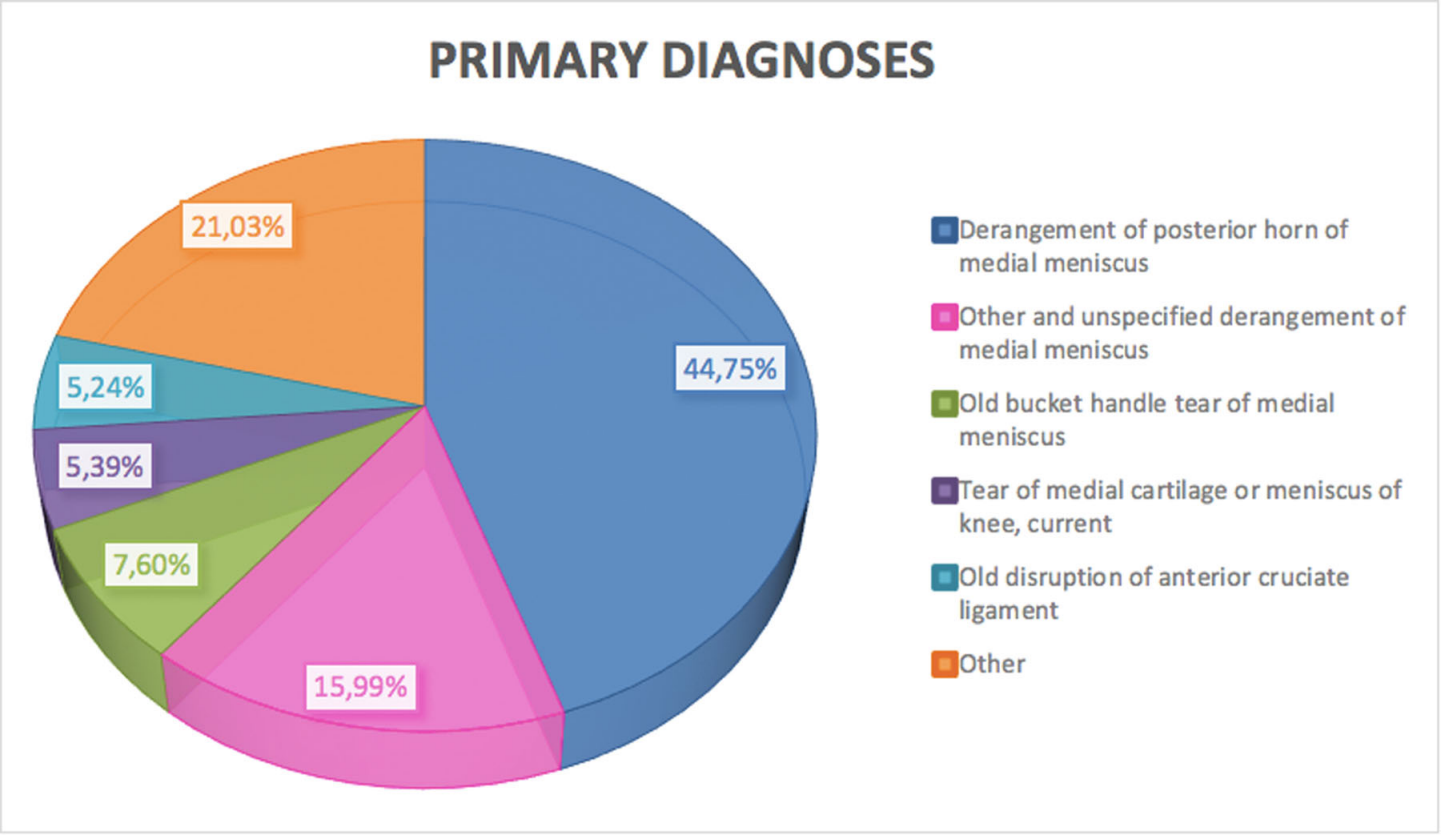
Main primary diagnoses (diagnoses are coded according to the International Classification of Diseases, Ninth Revision, Clinical Modification [ICD‐9‐CM]) requiring meniscectomy (≥15 years of age) from 2001 to 2016: the most frequently observed diagnosis code was derangement of the posterior horn of medial meniscus (44.75%, ICD code: 7172).

Over the 16 years, the main primary diagnosis codes were derangement of posterior horn of medial meniscus (44.75%, ICD code: 7172), Other and unspecified derangement of medial meniscus (15.99%, ICD code: 7173), Old bucket handle tear of medial meniscus (7.6%, ICD code: 7170), Tear of medial cartilage or meniscus of knee, current (5.39%, ICD code: 8360) and Old disruption of ACL (5.24%, ICD code: 71783) (Figure 5).

### Main primary procedures

**Figure 6:**
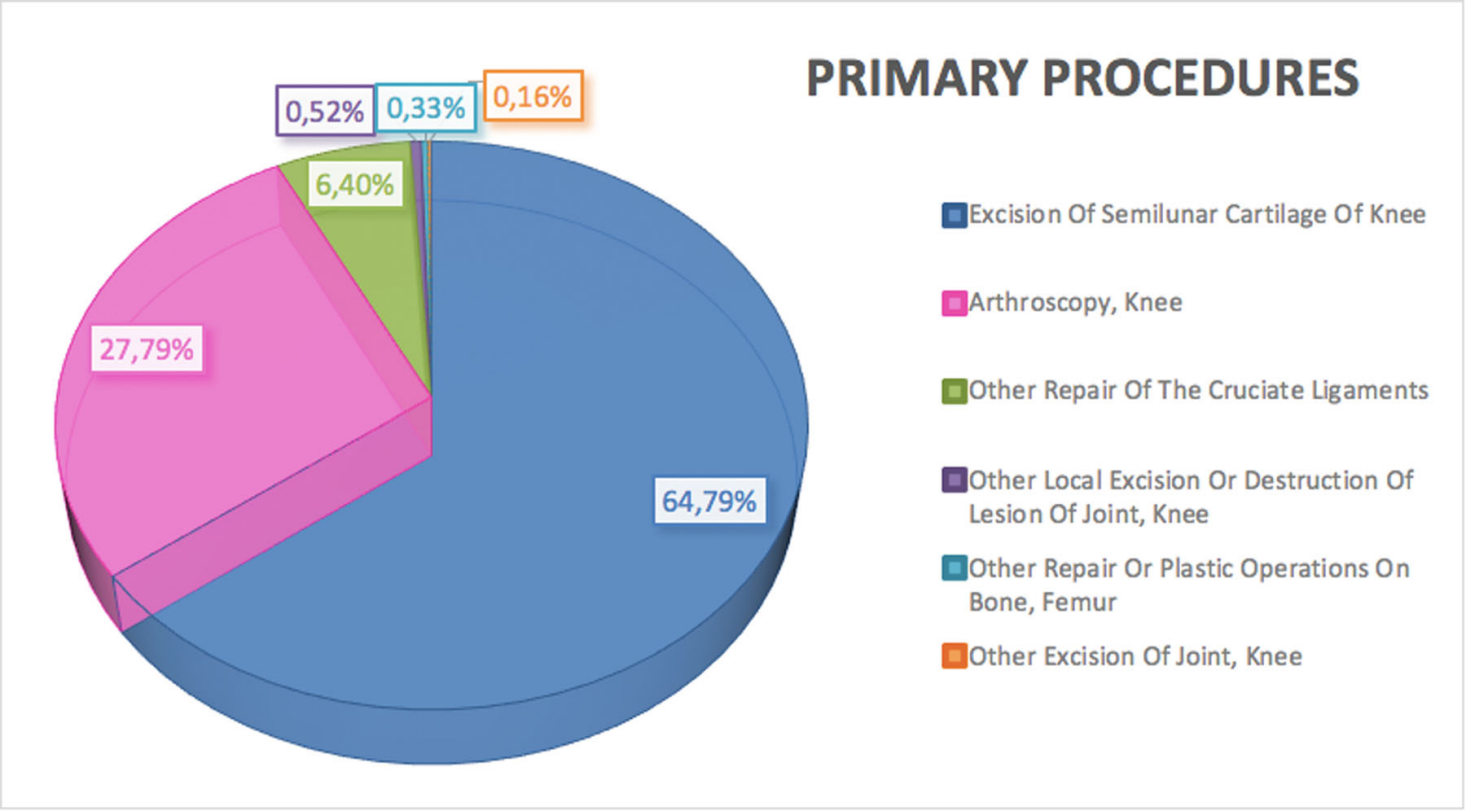
Main primary procedures for meniscectomy (procedures are coded according to the International Classification of Diseases, Ninth Revision, Clinical Modification [ICD‐9‐CM]) (≥15 years of age) from 2001 to 2016: the most frequently observed procedure code was Excision of Semilunar Cartilage of Knee (64.79%, ICD code: 806).

During the 16 years, the main primary surgical procedures were Excision of Semilunar Cartilage of Knee (64.79%, ICD code: 806), Arthroscopy, Knee (27.79%; ICD code: 8026), Other Repair of the Cruciate Ligaments (6.4%, ICD code: 8145), Other Local Excision or Destruction of Lesion of Joint, Knee (0.52%, ICD code: 8086), Other Repair or Plastic Operations on Bone, Femur (0.33%, ICD code: 7845) and Other Excision of Joint, Knee (0.16%, ICD code: 8096) (Figure 6).

### Economic Impact

To date, the average Italian hospital reimbursement for a meniscectomy ranges from EUR 2000 to 3500 for each hospital admission. Overall, between 2001 and 2016, a total cost ranging from EUR 181.861.375 to 318.257.406 has been calculated for meniscectomies. The annual average cost per 100,000 inhabitants was EUR 491.219 ± 122.148 with a range from EUR 291,500 ± 79.500 in 2016 to EUR 610,500 ± 166.500 in 2004 (Table 1). In Italy, reimbursement varies from region to region, hence explaining these ranges of excursion.

**Table 1:** Economic analysis of meniscectomy from 2001 to 2016 in Italy.

Year	Annual cost in EUR per 100,000 inhabitants (from minimum to maximum)		Annual average cost per 100,000 inhabitants ± DS in EUR
2001	404.000	707.000	555.500 ± 151.500
2002	424.000	742.000	583.000 ± 159.000
2003	428.000	749.000	588.500 ± 160.500
2004	444.000	777.000	610.500 ± 166.500
2005	438.000	766.500	602.250 ± 164.250
2006	432.000	756.000	594.000 ± 162.000
2007	434.000	759.500	596.750 ± 162.750
2008	434.000	759.500	596.750 ± 162.750
2009	418.000	731.500	574.750 ± 156.750
2010	338.000	591.500	464.750 ± 126.750
2011	312.000	546.000	429.000 ± 117.000
2012	278.000	486.500	382.250 ± 104.250
2013	256.000	448.000	352.000 ± 96.000
2014	242.000	423.500	332.750 ± 90.750
2015	222.000	388.500	305.250 ± 83.250
2016	212.000	371.000	291.500 ± 79.500

*Note*: Annual cost is presented from minimum to maximum in Columns 2 and 3. Annual average cost is presented in column 4.

## DISCUSSION

The most important finding of this study is the incidence of meniscectomy in the Italian population and its trend over the years. The current research is the first to provide Italian population‐based national trends for meniscectomy in the adult population. There are currently few epidemiological studies on this topic in the literature. Using a verified national registry, the amount of procedures performed in public and private hospitals in Italy was determined throughout the 15 years. According to the current analysis, there has been a marked decline in the incidence of meniscectomy in the adult population over the years. From 2001 to 2016, the number of procedures performed per 100,000 person years has almost halved. The decline observed is likely to be attributable to the increasing awareness of the orthopaedic community that meniscetomy is ineffective for relieving symptoms of OA and that outcomes of the procedure are less predictable in older patients, who are likely to have already developed OA. This concept is changing the modern approach to meniscal pathology in favour of conservative treatment, meniscal repair, or transplantation [2, 4, 23]. Similar results have been published in studies from other countries: in England, meniscectomy rate rose significantly between 1998 and 2013 (from 5.1/10,000 people to 14.9) before declining up to 2017 (to 12/10,000 people) [1]. According to Abrams et al., a rise in the number of isolated meniscal repair procedures was observed in the USA between 2005 and 2011 (11.4% increase in the total number of repairs) without observing an analogous rise in the number of meniscectomy [2]. Jacquet et al. reported in France declining meniscectomy rates from 19.80 per 10,000 people in 2005 to 15.77 per 10,000 people in 2017 (a reduction of 21.4%), whereas meniscus repair rates rising from 0.42 per 10,000 people in 2005 to 1.36 per 10,000 people in 2017 (a 320% increase) [14]. In the Italian paediatric population (patients between 0 and 14 years), the incidence trend of meniscectomy fell from 3.7 in 2001 to 2.8 in 2016, per 100,000 residents [23]. Literature seems to support the current global trend towards meniscus repair especially in younger patients, reducing the need for meniscectomy: results of the present study also suggest that Italian surgeons are aware of the more recent literature on this topic and that they are practicing evidence‐based medicine in this area.

Many orthopaedic diseases have different rates by gender [21, 22, 32]. The association between hormonal fluctuations and ACL injury in females in well known: studies indicate a possible link between menstrual cycle hormone levels and knee laxity or injury [12]. Sex hormone fluctuations have been shown to influence fibroblast proliferation and collagen synthesis, fundamental components of both ligaments and menisci [12]. Results from our study and from the literature suggest an opposite trend for meniscal pathology, with men more affected than women: according to data from the paediatric Italian population [23], also in the present study, males represented the majority of patients undergoing meniscectomy (68.2%). Similarly, meniscal repairs in the American population were more common in males [2]. Authors from the last study hypothesized that males are more prone to engaging high‐risk activities predisposing to knee injuries. Several studies confirmed that male gender is among the risk factors for meniscal lesions [34, 37]. As regards tear pattern, Jackson et al. showed that paediatric male patients had more vertical tears (31% vs. 21%) and posterior horn tears (71% vs. 60%), whereas female patients had a higher rate of degenerative tears (34% vs. 26%) [13]. Mai et al. showed that after meniscus surgery, females have a lower functional prognosis and quality of life than males [24]. Further studies are required in order to investigate biological factors that may contribute to these gender differences, including willingness to undergo surgery.

The age groups from 40 to 54 years demonstrated the highest incidence of meniscectomy in Italy over the study period. This is consistent with the literature which indicates that younger patients often have traumatic tears in the vascular zone ideal for meniscal repair, whereas older patients more frequently have tears that are best treated with partial meniscectomy [23]. Only 10%–30% of the adult peripheral meniscal tissue is vascular with poor healing potential [2]. Recently, the modern approaches to degenerative and traumatic meniscus lesions have been summarized [3, 18].

A decreasing trend in length of hospital stay was observed from 2001 to 2016 for meniscectomy. It is likely that it results shortening the length of hospital stay for all procedures to decrease costs.

The analysis of the primary procedure codes showed a higher rate of medial meniscectomy compared to lateral. The well‐documented ‘Brake stop mechanism’ of the medial meniscus posterior horn puts the medial meniscus to higher levels of shear stress in the ACL‐deficient knee [17, 25]. This area of the meniscus is therefore particularly vulnerable to tearing in the presence of ACL insufficiency, a frequent scenario in adult degenerated knees [17, 25].

The economic analysis of the present study indicates that the burden of meniscectomy is relevant in Italy. Studies have shown that the long‐term cost‐effectiveness and clinical results following meniscus repair outweigh those following meniscectomy [9, 38, 39]. For the healthcare system, meniscal repair costs more than meniscectomy: [35] this is probably due to implant costs, greater day‐of‐surgery costs, higher complication rates of meniscal repair than meniscectomy [7, 8]. However, in the long‐term period, meniscus repair becomes more cost‐effective because knee OA and consequent knee arthroplasties are less common [9]. A recent systematic review on the health‐economic evaluation of meniscus tear treatments concluded that meniscus repair is the most cost‐effective intervention for reparable meniscus tears [6]. Enhancing comprehension of charge and reimbursement patterns gives a chance for policymakers to improve resource management throughout the orthopaedics field [19].

The results of the present study should be considered in light of several limitations. First, all reported diagnoses and procedures are sourced from the ICD‐9‐CM. It is based on administrative records from multiple hospitals in a variety of regions, and these data are prone to error. Second, there are no outcome scores in this study because hospitalizations in Italy's healthcare system are anonymous and patients are not given an identifiable ID number. Therefore, patients who underwent more than one meniscectomy may have been counted more than once. Third, there can be differences between observers because ICD‐9 classification was performed by surgeons. A potential limitation of the economic analysis is given by the wide variability of reimbursements from region to region in Italy for the same procedure. Accordingly, it is not possible with our data to determine the exact costs afforded by the National Health Care System, but only to estimate an average between a minimum and a maximum value.

The operative treatment codes 806, 8026 and 8145 were considered in the present study. The 806 code stands for arthroscopic knee procedures. However, 8026 and 8145 are generic codes, used for both open and arthroscopic knee procedures. Therefore, this coding system did not allow us to differentiate between open and arthroscopic approaches used for meniscectomy performed during the study period. Moreover, 806 and 8026 codes do not specify if meniscus procedures were combined with other procedures on the knee joint. In contrast, 8145 code implies a concomitant procedure on the cruciate ligaments. The generic fashion of the ICD9‐CM codes resulted in the impossibility to determine the status of the ACL in patients undergoing meniscectomy.

The clinical relevance of this study is that the decreasing trend of meniscectomy in Italy, as observed in other countries, is in line with the emphasis on physician education regarding the significance of meniscus preservation and the rise in publications on the subject of the last decades. Surgeons must always take into account pros and cons when performing meniscal surgery, whereas patients should be advised of the potential sequelae and the effects on meniscal outcomes of this type of surgery.

## CONCLUSIONS

Overall, 1,454,891 Meniscectomies were performed in the study period between 2001 and 2016. Medial meniscectomy was more common than lateral. The age groups from 40 to 54 years showed the highest rates of meniscectomy. This analysis showed that the number of meniscectomies performed in Italy in the adult population has almost halved over the study period. The results of the present study seem to be in line with recent orthopaedic recommendations, indicating that meniscal surgery in the setting of an arthritic knee is less predictable and should be performed sparingly and that meniscal preservation (meniscal repair and conservative treatment) is becoming more popular. Results of the present study in the Italian population seem to reflect how the clinical evidence basis affects surgical technique selection.

Moreover, the economic burden of meniscectomy is relevant in Italy with an estimated expenditure from EUR 181.861.375 to 318.257.406 between 2001 and 2016. Enhancing comprehension of charge and reimbursement patterns gives a chance for policymakers to improve resource management throughout the orthopaedics field.

## AUTHOR CONTRIBUTIONS

**Umile Giuseppe Longo, Alessandro Mazzola, Robert Marx**: Manuscript preparation; study design; database interpretation and manuscript revision. **Umile Giuseppe Longo, Alessandro Mazzola, Ilaria Piergentili, Sergio De Salvatore**: Manuscript preparation; database interpretation and statistical analysis. **Alessandro Mazzola, Ilaria Piergentili, Marco E. Cardinale, Sergio De Salvatore**: Manuscript preparation; figures preparation; study design. **Umile Giuseppe Longo, Alessandro Mazzola, Marco E. Cardinale**: Manuscript preparation and database interpretation. **Rocco Papalia, Umile Giuseppe Longo, Alessandro Mazzola, Robert Marx**: Study design; manuscript revision. All authors read and approved the final article.

## CONFLICT OF INTEREST STATEMENT

The authors declare no conflict of interest.

## ETHICS STATEMENT

The Institutional Review Board of Campus Bio‐Medico University of Rome ruled that no formal ethics approval was required in this particular case.
